# Impact of Sub-Inhibitory Concentrations of Amoxicillin on *Streptococcus suis* Capsule Gene Expression and Inflammatory Potential

**DOI:** 10.3390/pathogens5020037

**Published:** 2016-04-19

**Authors:** Bruno Haas, Daniel Grenier

**Affiliations:** 1 Groupe de Recherche en Écologie Buccale (GREB), Faculté de Médecine Dentaire, Université Laval, Quebec City, QC G1V 0A6, Canada; brunohaas67@gmail.com; 2 Centre de Recherche en Infectiologie Porcine et Avicole (CRIPA), Fonds de Recherche du Québec—Nature et Technologies (FRQNT), Saint-Hyacinthe, QC J2S 2M2, Canada

**Keywords:** *Streptococcus suis*, amoxicillin, virulence, antibiotic, sub-inhibitory concentration, cytokine, inflammatory response

## Abstract

*Streptococcus suis* is an important swine pathogen and emerging zoonotic agent worldwide causing meningitis, endocarditis, arthritis and septicemia. Among the 29 serotypes identified to date, serotype 2 is mostly isolated from diseased pigs. Although several virulence mechanisms have been characterized in *S. suis*, the pathogenesis of *S. suis* infections remains only partially understood. This study focuses on the response of *S. suis* P1/7 to sub-inhibitory concentrations of amoxicillin. First, capsule expression was monitored by qRT-PCR when *S. suis* was cultivated in the presence of amoxicillin. Then, the pro-inflammatory potential of *S. suis* P1/7 culture supernatants or whole cells conditioned with amoxicillin was evaluated by monitoring the activation of the NF-κB pathway in monocytes and quantifying pro-inflammatory cytokines secreted by macrophages. It was found that amoxicillin decreased capsule expression in *S. suis*. Moreover, conditioning the bacterium with sub-inhibitory concentrations of amoxicillin caused an increased activation of the NF-κB pathway in monocytes following exposure to bacterial culture supernatants and to a lesser extent to whole bacterial cells. This was associated with an increased secretion of pro-inflammatory cytokines (CXCL8, IL-6, IL-1β) by macrophages. This study identified a new mechanism by which *S. suis* may increase its inflammatory potential in the presence of sub-inhibitory concentrations of amoxicillin, a cell wall-active antibiotic, thus challenging its use for preventive treatments or as growth factor.

## 1. Introduction

*Streptococcus suis* (*S. suis*) is considered one of the most important swine pathogens as it is responsible for substantial economical losses in the swine industry worldwide. The bacterium is naturally found in the respiratory and digestive tracts of swine and its natural ecological niche is believed to be the palatine tonsils [1]. To date, 29 serotypes of *S. suis* have been identified, based on the composition of the capsular polysaccharides [2,3]. Serotype 2 is considered the most virulent among the species as it is the most common serotype isolated from diseased pigs worldwide. *S. suis* is known to cause endocarditis, pneumonia and arthritis, but the most life-threatening infection is meningitis that can leave permanent sequelae such as hearing loss, and streptococcal-like toxic shock syndrome that can lead to death within hours post-infection [4]. *S. suis* is also a zoonotic agent, mostly in Asia where two major outbreaks occurred in 1998 and 2005 resulting in over 50 deaths [5]. Sporadic human cases are also described at the world scale with symptoms similar to those occurring in swine [6].

As for any bacterial infection, antibiotics represent an efficient and affordable way to control *S. suis* infections since vaccine development remains challenging due to the high genetic and phenotypic variability within the species [7]. The most commonly used antibiotics to treat *S. suis* infections are β-lactams (penicillin G, ceftiofur, amoxicillin), aminoglycosides (gentamycin), phenicols (florfenicol) and fluoroquinolones [8]. However, the selective pressure caused by the systematic use of antibiotics (either in therapeutic or preventive treatments as well as growth factors) is known to be correlated with the apparition of resistance against these antimicrobial agents [9,10]. Furthermore, it has been shown in many bacterial species that the use of antibiotics at concentrations lower than the minimal inhibitory concentration (MIC) (known as sub-inhibitory concentrations) can modulate bacterial virulence [11]. On the one hand, decreased virulence or virulence factor gene expression have been observed in both gram-negative and gram-positive bacteria, especially with antibiotics targeting the protein synthesis [12,13,14,15]. On the other hand, under the action of cell wall-targeting antibiotics, the expression of virulence-associated genes can be upregulated resulting in an increased virulence of bacteria [16]. Interestingly, Shen *et al.* investigated the effect of vancomycin, a glycopeptide type antibiotic that targets the cell wall synthesis in gram-positive bacteria, on the gram-negative bacterium *Pseudomonas aeruginosa*, and found that sub-inhibitory concentrations of this antibiotic increase virulence factor gene expression [17].

Amoxicillin is a β-lactam antibiotic of moderate spectrum belonging to the penicillin class. This bacteriolytic antibiotic prevents the cross-linkage between the peptidoglycan linear chains, thus inhibiting cell-wall synthesis in gram-positive bacteria. In the present study, we hypothesized that the use of sub-inhibitory concentrations of amoxicillin can modulate the biological properties of *S. suis*. First, the effect of sub-inhibitory concentrations of amoxicillin on the expression of *cps2J* gene has been tested. Then, the modulation of the pro-inflammatory potential of *S. suis* whole cells or culture supernatants by sub-inhibitory concentrations of amoxicillin has been analyzed by monitoring NF-κB activation in monocytes and quantifying pro-inflammatory cytokine secretion by macrophages.

## 2. Results

### 2.1. Determination of MIC of Amoxicillin for S. suis

*S. suis* P1/7 has been selected in this study as it is a reference strain of *S. suis* serotype 2 used by numerous groups worldwide. Following a 24-h incubation of *S. suis* P1/7 in THB at 37 °C, growth was found to be completely inhibited in the presence of 12.5 ng/mL of amoxicillin. According to these observations, the MIC of amoxicillin for *S. suis* P1/7 has been established at 12.5 ng/mL.

### 2.2. S. suis Capsule Expression

**Figure 1:**
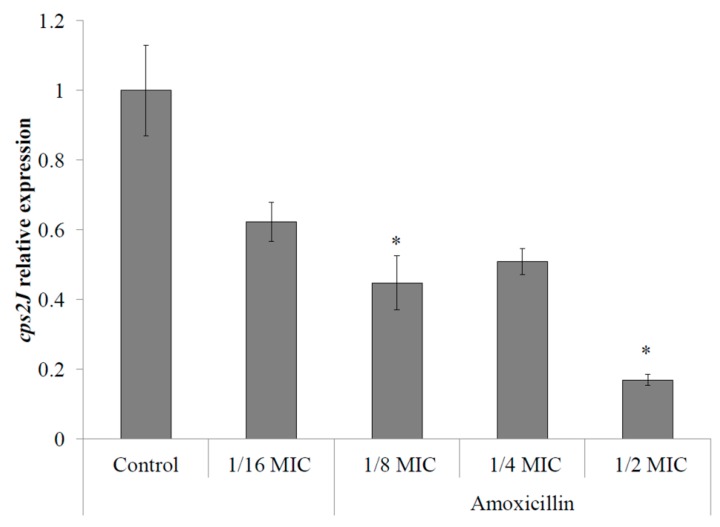
Relative expression of the capsule gene *cps2J* by *S. suis* P1/7 in the presence of sub-inhibitory concentrations of amoxicillin. Control: no antibiotics. *: *p* < 0.01.

The response of *S. suis* to sub-inhibitory concentrations of amoxicillin in regard to capsule expression has been quantified by monitoring *cps2J* gene expression by qPCR following a 6-h incubation. The expression of *cps2J* by *S. suis* P1/7 has been found to be dose-dependently down-regulated in the presence of sub-inhibitory concentrations of amoxicillin. Expression of *cps2J* showed a significant decrease of 80% and 50% in the presence of 1/2 and 1/8 MIC of amoxicillin (6.25 and 1.5625 ng/mL), respectively, when compared with the control assay (no antibiotics) (Figure 1).

### 2.3. Visualization of S. suis Capsule by Transmission Electron Microscopy

**Figure 2:**
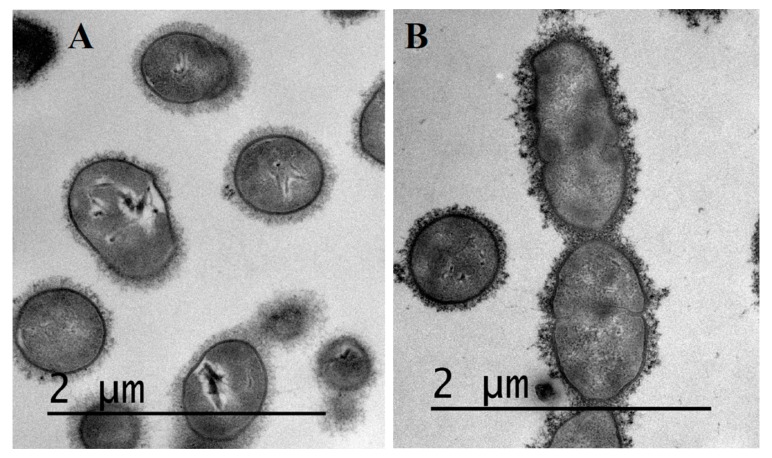
Transmission electron microscopy of overnight cultures of *S. suis* P1/7 grown in THB alone (**A**) or THB supplemented with 1/2 MIC of amoxicillin (**B**).

Transmission electron microscopy was performed on bacteria grown overnight in THB supplemented or not with 1/2 MIC of amoxicillin to visualize *S. suis* capsule integrity under these conditions. As seen on Figure 2, no significant differences could be observed in terms of capsule thickness between overnight cultures of *S. suis* P1/7 in THB alone (Figure 2A) and THB supplemented with 1/2 MIC of amoxicillin (Figure 2B). However, in the presence of amoxicillin, *S. suis* capsule appears heterogeneous, with dense granular structures as compared to conditions without antibiotics.

### 2.4. Pro-Inflammatory Potential of S. suis

The pro-inflammatory potential of *S. suis* whole cells and culture supernatants was investigated following growth in the presence of amoxicillin at 1/2, 1/4, 1/8, and 1/16 MIC. It was also observed that when the incubation period of *S. suis* cultivated in the presence of amoxicillin at MIC (12.5 ng/mL) was extended to 40 h, a reduced growth (40% to 50% of control) occurred. Therefore, cells and the culture supernatant from this condition were also tested.

#### 2.4.1. Activation of the NF-κB Pathway in Monocytes

**Figure 3:**
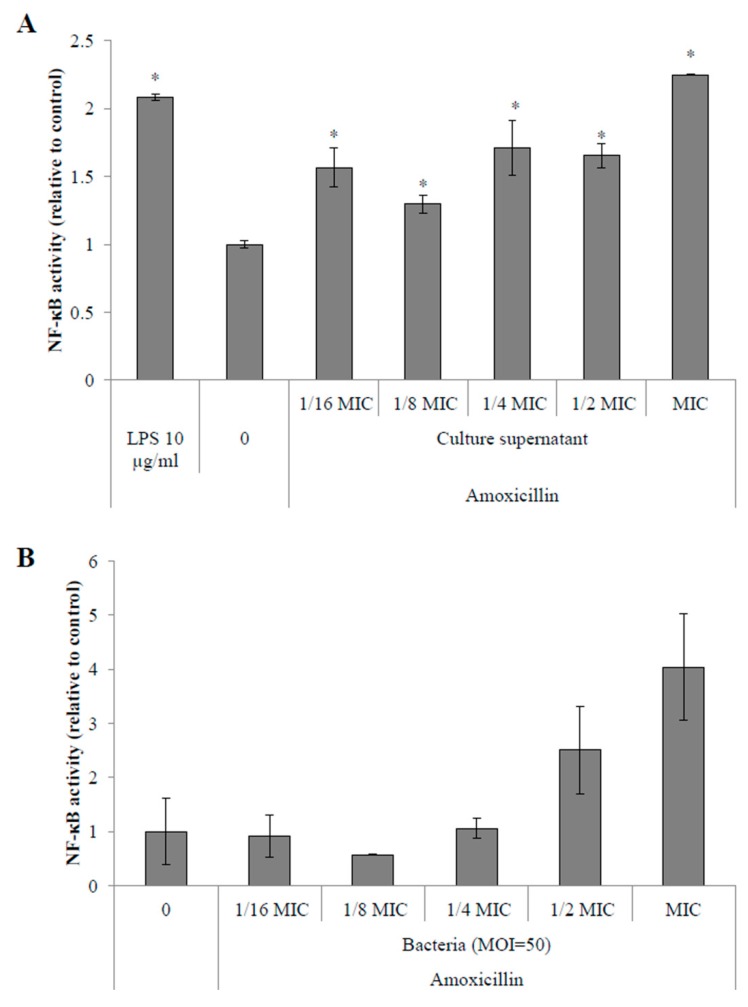
Activation of NF-κB signaling pathway in monocytes stimulated with culture supernatants (**A**) or *S. suis* P1/7 whole cells (**B**) conditioned in sub-inhibitory concentrations of amoxicillin. *Escherichia coli* LPS was used as a positive control. 0: no antibiotic. *: *p* < 0.05.

Stimulations of monocytes with filtered, pH-adjusted culture supernatants of *S. suis* P1/7 showed an overall increase of NF-κB activation in monocytes. Activation of the NF-κB pathway was increased by 30% to 70% when monocytes were stimulated with supernatants of *S. suis* P1/7 from cultures in the presence of 1/2 to 1/16 MIC of amoxicillin (Figure 3A). Moreover, when amoxicillin was used at the MIC, NF-κB activation by the culture supernatant was found to be 2.25-fold higher than a control culture supernatant (without antibiotic).

Stimulation of monocytes with whole bacteria also showed an increased activation of the NF-κB pathway. More specifically, when bacteria were conditioned with ampicillin at MIC (12.5 ng/mL) and 1/2 MIC (6.25 ng/mL), a 4- and 2.5-fold increase of NF-κB activation was observed. However, these results were not significantly different (< 0.05) from control bacteria when Student’s *t*-test was applied (Figure 3B).

#### 2.4.2. Pro-Inflammatory Cytokine Secretion by Macrophages

**Figure 4:**
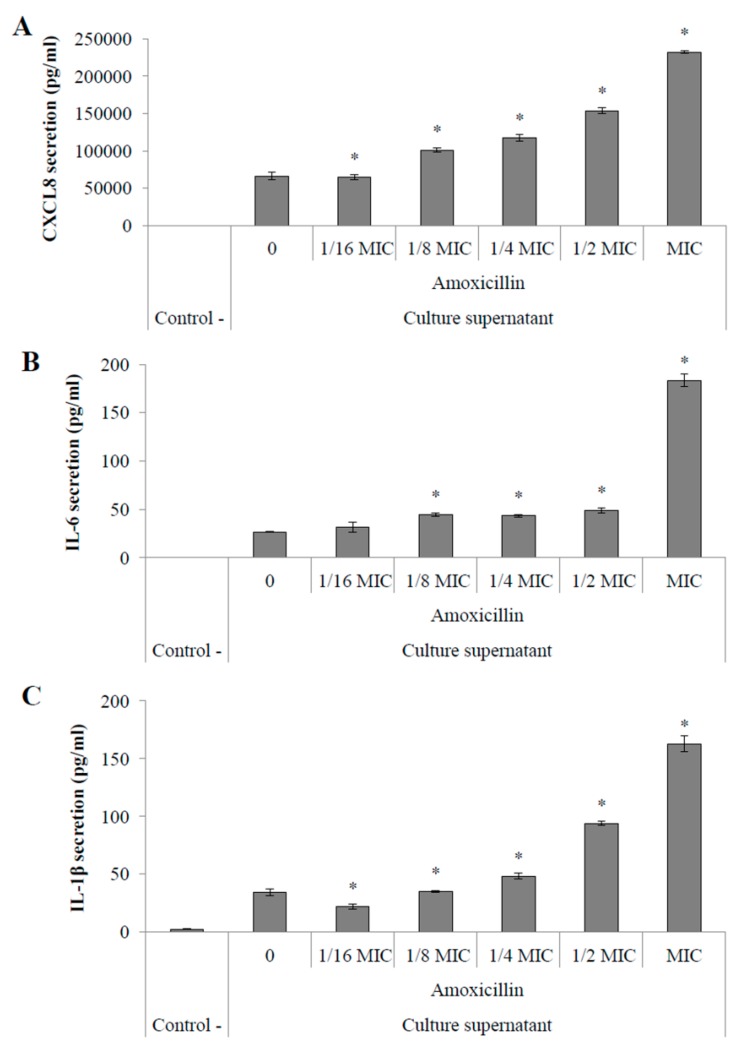
Secretion of pro-inflammatory cytokines CXCL8 (**A**); IL-6 (**B**) and IL-1β (**C**) by macrophages stimulated with culture supernatants of *S. suis* P1/7 conditioned with amoxicillin. Control - : no stimulation. 0: no antibiotic. *: *p* < 0.05.

**Figure 5:**
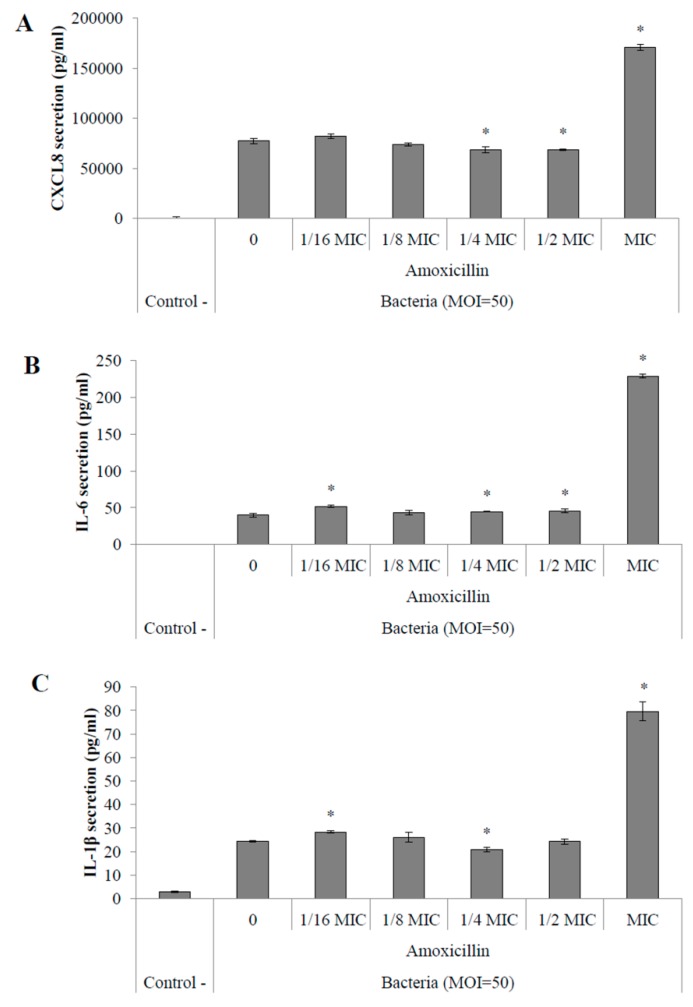
Secretion of pro-inflammatory cytokines CXCL8 (**A**); IL-6 (**B**) and IL-1β (**C**) by macrophages stimulated with *S. suis* P1/7 whole cells (MOI = 50) conditioned with amoxicillin. Control - : no stimulation. 0: no antibiotic. *: *p* < 0.05.

Stimulations of macrophages with supernatants obtained from the same cultures used in monocyte stimulations showed an amoxicillin MIC-dependent increased secretion of CXCL8, IL-6 and IL-1β (Figure 4A–C, respectively). When stimulated with culture supernatants of *S. suis* P1/7 grown in the presence of 12.5 ng/mL amoxicillin, macrophages secreted 3.5-fold more CXCL8 (Figure 4A), 6.8-fold more IL-6 (Figure 4B) and 4.7-fold more IL-1β (Figure 3C) than stimulations with a control supernatant (without antibiotics). Stimulation with a supernatant from a culture in the presence of 1/2 MIC of amoxicillin (6.25 ng/mL) resulted in an increased secretion of CXCL8, IL-6, and IL-1β by macrophages by 2.3-, 1.8- and 2.7-fold, respectively. Moreover, an increased secretion of CXCL8, IL-6, and IL-1β by macrophages was only observed with *S. suis* P1/7 whole cells recovered from a culture in the presence of amoxicillin at MIC (Figure 5A–C, respectively).

## 3. Discussion

Since their discovery, antibiotics have been widely used to treat bacterial infections with high efficiency. When used properly, at the appropriate concentrations and for the right target, these molecules can kill bacterial pathogens or inhibit their growth. However, if used at concentrations lower than the MIC, antibiotics can modulate bacterial fitness as well as gene expression [18]. For example, cell wall-targeting antibiotics were shown to inhibit capsule formation in *Streptococcus pneumoniae* [19]. Furthermore, the massive use of antibiotics at sub-inhibitory concentrations in the animal agriculture either as preventive treatments or as growth factors has lead to the emergence of bacterial strains resistant to one or more of these molecules [20]. It is thus of utmost importance to understand the bacterial behavior in response to a stress induced by sub-inhibitory concentrations of antibiotics. This study focused on the impact of sub-inhibitory concentrations of amoxicillin on capsule expression and inflammatory potential of the swine pathogen S*. suis*. It has been previously reported that cell wall-targeting antibiotics can induce an increased expression of virulence factors in both gram-negative and gram-positive bacteria, thus promoting their virulence [16,17]. Regarding *S. suis*, a stress induced by sub-inhibitory concentrations of erythromycin was found to reduce biofilm formation through downregulation of numerous surface proteins as well as mediators of quorum-sensing [21].

The sialic acid-rich capsule is considered as one of the most important virulence factor in *S. suis* as its loss causes an increased pro-inflammatory potential of the bacterium and reduces its resistance to macrophage-mediated phagocytosis and the action of some antibiotics [22,23]. The *cps2J* gene is responsible for the side chain formation of the capsule and also represents a molecular marker for serotypes 2 and 1/2 [24,25]. When *S. suis* was incubated for 6 h in the presence of sub-inhibitory concentrations of amoxicillin, the *cps2J* gene was down-regulated, suggesting modifications in the capsule structure. Transmission electron microscopy on overnight cultures of *S. suis* P1/7 in THB supplemented or not with 1/2 MIC of amoxicillin did not show a significant difference in terms of capsule thickness, however, in the presence of 1/2 MIC of amoxicillin, the capsule was found to display a heterogeneous structure when compared to normal conditions. This is in accordance with the structural role of *cps2J* in capsule production. The granular structure of the capsule found in the presence of amoxicillin could result in an increased porosity as side chains would not be properly formed, thus exposing surface proteins. On the one hand, the loss of capsule or its architectural modifications in *S. suis* has been associated with an increased susceptibility to phagocytosis by macrophages and neutrophils [26,27] and a decreased virulence in both pig and mouse models [22,28,29]. Such a phenomenon would be beneficial for the host as it could facilitate clearance of the pathogen during infection. On the other hand, it has also been observed that unencapsulated strains of *S. suis* display a higher capacity of adherence to and invasion of HEp-2 epithelial cells suggesting that down-regulation of *S. suis* capsule expression during the early steps of infection could increase adherence of bacteria to host cells by uncovering surface adhesins [30]. More recently, the loss of capsule expression in *S. suis* strains was correlated with a higher potency to induce endocarditis in swine [31]. Furthermore, the pro-inflammatory potential of unencapsulated mutants of *S. suis* was found to be increased compared to their parental strains [23]. According to these observations and our findings, ones could hypothesize that *S. suis* is able to modify the structure of its capsule in response to sub-inhibitory concentrations of amoxicillin and therefore becomes more invasive within the host. The higher inflammatory potential of these strains could lead to a dysregulation of the host immune response thus causing septicemia. Since *S. suis* virulence is multifactorial, expression of other genes coding for virulence-associated factors could be modulated by the presence of sub-inhibitory concentrations of antibiotics. Further research should be carried on concerning the expression of other virulence factor genes.

Since *S. suis cps2J* expression was found to be significantly down-regulated in the presence of sub-inhibitory concentrations of amoxicillin, by extrapolation of the microscopic observations, it can be suggested that the capsule structure is likely affected under these conditions. Furthermore, as previously stated, loss or changes in the capsule structure generally leads to an increased pro-inflammatory potential suggesting a higher capacity to dysregulate the host immune system [23]. Streptococcal septic shock can be caused by such a deregulation as it is the result of an overstimulation of the host immune system [32]. In order to evaluate the inflammatory potential of *S. suis* in response to an amoxicillin stress, stimulation of monocytes and macrophages were performed with both whole cells and culture supernatants following a 40-h incubation with sub-inhibitory concentrations of the antibiotic. Interestingly, extending incubation to 40 h allowed the growth of *S. suis* even with 12.5 ng/mL amoxicillin, which corresponds to the MIC for a 24-h culture in THB. However, even though growth was observed under this condition, it was found to be only 40%–50% of that obtained without antibiotics (data not shown). NF-κB is an important transcriptional activator of cytokine gene expression and highly regulates pro-inflammatory cytokine production [33]. The use of amoxicillin at 1/2 to 1/16 MIC resulted in an increased activation of the NF-κB pathway in monocytes when stimulated with culture supernatants suggesting an increased secretion of pro-inflammatory activators by *S. suis* in response to these conditions. An increased activation of NF-κB was also found when monocytes were stimulated with whole cells of *S. suis* conditioned with amoxicillin at MIC and 1/2 MIC. The stimulation of PMA-differentiated monocytes with *S. suis* supernatants resulted in an increased secretion of pro-inflammatory cytokines such as CXCL8, IL-6 and IL-1β, especially from cultures in the presence of amoxicillin at MIC to 1/8 MIC. Furthermore, the secretion of these mediators was found to be dose-dependent on the concentration of amoxicillin used in the bacterial cultures. Again, similar stimulations using *S. suis* whole cells conditioned with sub-inhibitory concentrations of amoxicillin showed a high increase in pro-inflammatory cytokine secretion by macrophages only at MIC. These results are in accordance with a previous study that showed that macrophages display an increased secretion of pro-inflammatory cytokines in response to stimulations with *S. pneumoniae* conditioned with sub-inhibitory concentrations of β-lactams compared to control cultures [34].

Taken altogether, this study brought evidence that the use of antibiotics at lower dosage than recommended could lead to an increased immunogenicity of *S. suis* suggesting a higher virulence as a response to such a stress. Indeed, *S. suis* infections can lead to streptococcal toxic shock-like syndrome characterized by a high secretion of pro-inflammatory cytokines [35,36]. Previous studies showed that increased immunogenicity of *S. suis* was correlated with higher secretion of pro-inflammatory cytokines by macrophages resulting from the recognition of the pathogen by mainly TLR-2 and -6, thus activating the NF-κB pathway [37,38]. The use of antibiotics as growth promoters dates back to the 1950s and generally uses low dosage of antibiotics, below the MIC. Generally, antibiotics used for preventive treatments or as growth factors are added to the water or food [39]. Therefore, it is most likely that levels of antibiotics encountered by pathogens in the animal circulatory system or in its organs are below the MIC. To our knowledge, this study shows for the first time the resulting response of *S. suis* to stress induced by sub-inhibitory concentrations of amoxicillin and therefore the importance of proper and careful use of antimicrobial agents in the industry.

## 4. Materials and Methods

### 4.1. Bacteria and Growth Conditions

*S. suis* P1/7 (serotype 2, sequence type 1) was routinely grown in Todd-Hewitt Broth (THB, Beckton, Dickinson and Co., Sparks, MD, USA) at 37 °C supplemented or not with amoxicillin (Sigma-Aldrich Canada Ltd., Oakville, ON, Canada).

### 4.2. Determination of Minimal Inhibitory Concentration (MIC) of Amoxicillin on S. suis

Two-fold serial dilutions of amoxicillin in THB (from 100 ng/mL) were prepared. An overnight preculture of *S. suis* P1/7 at 37 °C in THB was used to inoculate 10 mL of amoxicillin-supplemented THB (OD_660_ final = 0.05). Cultures were grown for 24 h at 37 °C and bacterial growth was monitored by measuring absorbance at 660 nm.

### 4.3. S. suis Capsule Expression

An overnight preculture of *S. suis* P1/7 in THB at 37 °C was used to inoculate 10 mL of amoxicillin-supplemented THB (two-fold serial dilutions from MIC [12.5 ng/mL] to 1/16 MIC). Cultures were incubated for 6 h at 37 °C prior to RNA extraction using the Qiagen RNeasy minikit (Qiagen Inc., Mississauga, ON, Canada), according to the manufacturer’s recommendations. RNA quality was tested using the Experion RNA StdSens Analysis kit (Bio-Rad Laboratories, Mississauga, ON, Canada). Purity and quantity of the RNA were monitored using Nanodrop (Thermo Fisher Scientific, Wilmington, DE, USA). Samples were then diluted in nuclease-free water to a final concentration of 100 ng/µL and cDNA were produced using the iScript R-T Supermix for RT-qPCR (Bio-Rad Laboratories) following the manufacturer’s recommendations. Gene expression of *S. suis* capsule (*cps2J*) was recorded using the CFX96 Real-Time System C1000 Thermal Cycler (Bio-Rad Laboratories) according to the manufacturer’s protocols, using the 16S rRNA as internal control for data normalization, a well-known control used for gene expression quantification in *S. suis* [40]. Primers (Life Technologies Inc., Burlington, ON, Canada) and expected amplicon sizes are listed in Table 1. Primers specificity was tested by analyzing melting curves and migration of PCR products on 1% agarose gel for 45 min at 100 V. Amplicons migrated to their expected size of 123 bp (16S rRNA) and 198 bp (*cps2J*) (data not shown).

### 4.4. Visualization of S. suis Capsule by Transmission Electron Microscopy

Five milliliters of overnight cultures of *S. suis* P1/7 at 37 °C in THB alone or in THB supplemented with 1/2 MIC of amoxicillin were centrifuged 5 min at 10,000 g, and pellets were washed three times with 50 mM phosphate-buffered saline (PBS, pH 7.2). Bacteria were suspended in 0.1 M sodium cacodylate buffer (pH 7) containing 5% glutaraldehyde and 0.15% ruthenium red. Samples were then treated with 1 mg/mL of polycationic ferritin and were processed as previously described by Vanrobaeys *et al.* [41]. Thin sections were prepared and observed using a JEOL 1230 transmission electron microscope (JEOL USA Inc., Peabody, MA, USA) at an accelerating voltage of 80 kV.

### 4.5. Analysis of the Pro-Inflammatory Potential of S. suis Conditioned with Sub-Inhibitory Concentrations of Amoxicillin

*S. suis* P1/7 was grown in THB supplemented with amoxicillin (two-fold serial dilutions from MIC to 1/16 MIC) for 40 h at 37 °C. Bacteria and culture supernatants were then collected by centrifugation (5 min at 10,000 *g*). Bacteria were resuspended in RPMI-1640 medium (Life Technologies Inc.) supplemented with 1% heat-inactivated fetal bovine serum (FBS) and 100 µg/mL penicillin-streptomycin. Culture supernatants were filtered through a 0.22 µm pore-size membrane filter, pH was adjusted to 7 by addition of 1 M NaOH and then diluted in RPMI-1640 medium supplemented with 1% heat inactivated FBS and 100 µg/mL penicillin-streptomycin (1:2/v:v). These preparations were later used for monocyte and macrophage stimulations.

#### 4.5.1. Activation of the NF-κB Pathway in Monocytes

The human monoblastic leukemia cell line U937-3XκB-LUC (U937 cells stably transfected with a construct containing 3 NF-κB binding sites from the Ig κ light chain promoter coupled with the gene encoding the firefly luciferase (3X-κB-luc) [42]) was used to monitor NF-κB activation. This cell line was kindly provided by Dr. Rune Blomhoff (University of Oslo, Oslo, Norway). Cells were routinely grown at 37 °C in a 5% CO_2_ atmosphere in RPMI-1640 medium supplemented with 10% heat-inactivated FBS, 100 µg/mL penicillin-streptomycin, and 75 µg/mL hygromycin B (Sigma-Aldrich Canada Ltd.). The monocytes were harvested by centrifugation (5 min at 270 g) and resuspended in RPMI-1640 supplemented with 1% heat-inactivated FBS and 100 µg/mL penicillin-streptomycin at 2.10^6^ cells/mL. Fifty microliters of the cell suspensions were seeded into a black wall and black bottom 96-well microplate (Greiner Bio-One GmbH, Frickenhausen, Germany). Fifty microliters of the previous bacterial suspensions and diluted culture supernatants were added to the cells in order to reach a multiplicity of infection (MOI) of 50 for bacteria or a 1:4 (v:v) dilution for the culture supernatants. Cells were stimulated during 6 h at 37 °C in a 5% CO_2_ atmosphere. *Escherichia coli* lipopolysaccharide (LPS) (10 µg/mL) was used as a positive control whereas bacteria and culture supernatants of *S. suis* P1/7 grown in THB without antibiotics were used as reference control for data comparison. NF-κB activation was monitored using the Bright-Glo Luciferase Assay System (Promega, Madison, WI, USA) by adding 100 µL of luciferase substrate to the wells at room temperature. Luminescence was recorded using the luminometer option of the Synergy 2 microplate reader (BioTek Instruments, Winooski, VT, USA) within 3 min following the addition of the substrate.

#### 4.5.2. Quantification of Pro-Inflammatory Cytokine Secretion by Macrophages

The human monoblastic leukemia U937 cell line was cultivated in RPMI-1640 supplemented with 10% heat-inactivated FBS and 100 µg/mL of penicillin-streptomycin at 37 °C under a 5% CO_2_ atmosphere. The monocytes (2.10^5^ cells/mL) were differentiated into macrophage-like cells by incubation with 10 ng/mL of phorbol myristate acetate (PMA) for 24 h. The culture medium was then replaced with fresh medium and adherent macrophages were incubated for an additional 24 h. Cells were then collected by gentle scraping, counted and centrifuged 5 min at 270 g and resuspended in RPMI-1640 medium supplemented with 10% heat inactivated FBS and 100 µg/mL penicillin-streptomycin to a final concentration of 10^6^ cells/mL. Two mL of the cell suspension were added into wells of a 6-well cell culture microplate, and a 4 h incubation at 37 °C under a 5% CO_2_ atmosphere allowed the cells to adhere to the wells. The medium was then aspirated prior to the addition of bacteria or culture supernatants used in the monocyte stimulations. RPMI-1640 supplemented with 1% heat-inactivated FBS and 100 µg/mL penicillin-streptomycin was added to the wells in order to reach a final MOI of 50 and a 1/4 dilution of bacterial culture supernatants. A negative control consisting of only cell culture medium (RPMI-1640 supplemented with 1% heat-inactivated FBS and 100 µg/mL penicillin-streptomycin) was performed as well as stimulations with bacteria or culture supernatants of *S. suis* P1/7 grown in THB without amoxicillin for data comparison. Following a 24-h incubation at 37 °C in a 5% CO_2_ atmosphere, culture supernatants were collected and quantification of the pro-inflammatory cytokines interleukin (IL)-8 (CXCL8), IL-6 and IL-1β was performed by enzyme-linked immunosorbent assay (ELISA) kits following the manufacturer’s protocols (eBioscience Inc., San Diego, CA, USA).

In both NF-κB activation and pro-inflammatory cytokine quantification assays, data from stimulations of monocytes and macrophages with THB supplemented with amoxicillin alone were subtracted to data from stimulations with the corresponding culture supernatants in order to eliminate a possible background secretion resulting from the effect of amoxicillin on monocytes or macrophages.

### 4.6. Statistical Analysis

All assays were performed in triplicate, and the means ± standard deviations were calculated. Assays were done in three independent replicates and a representative set of data is reported. Differences were analyzed for statistical significance using the Student’s *t*-test and were considered significant at *p* < 0.01 for the gene expression and *p* < 0.05 for the stimulation assays.

## Figures and Tables

**Table 1:** Primers used for the determination of capsule expression in *S. suis* P1/7.

Gene	Primer Sequence (5'-3')	Expected Amplicon Size (bp)
16S rRNA	Forward: TAGGGTTTCTCTTCGGAGCATCG	123
	Reverse: AACTGAATGATGGCAACT	
*cps2J*	Forward: AGAGTGTTTAGATAGCATTATTTC	198
	Reverse: TAATTTGCTGTGCTATTTTTGATAC	

## Abbreviations

The following abbreviations are used in this manuscript:

cDNA	complementary deoxyribonucleic acid
CXCL8	interleukin-8
FBS	fetal bovine serum
IL	interleukin
LPS	lipopolysaccharide
MIC	minimal inhibitory concentration
MOI	multiplicity of infection
NF-κB	nuclear factor-κB
PBS	phosphate-buffered saline
PCR	polymerase chain reaction
PMA	phorbol myristate acetate
qRT-PCR	quantitative reverse transcription polymerase chain reaction
RNA	ribonucleic acid
RPMI-1640	Roswell Park Memorial Institute medium-1640
THB	Todd-Hewitt broth
